# 

**Published:** 1903-12-26

**Authors:** 


### CONTENTS.

**The Opportunities of the Wealthy** - - - - 219

**The Medical Care of Children in Elementary Schools** - - - - - - - - - - - - 220

**Annotations** - - - - - - - - - - - - - 221

**Notes and News** - - - - - - - - - - - 222

**Hospital Clinics and Medical Progress—**

What We Owe to Experiments on Animals - - - - - 223

Aneurysm of the Abdominal Aorta - - - - - - 224

The Relation of Mild Types of Diphtheria to the Public Health - 225

Syphilitic Joint Disease - - - - - - - - 226

Diseases of the Heart and Circulation - - - - - 226

Progress in Diabetes - - - - - - - - - - 227

Progress in Cancer - - - - - - - - - - 228

**New Appliances and Things Medical** - - - 229

**The Book World of Medicine and Science** - - 230

**Hospital Administration—**

The League of Mercy - - - - - - - - - 231

Royal Victoria Hospital, Belfast - - - - - - - 232

**The Student of Medicine** - - - - - - - - 234

*NURSING SECTION.*

**Notes on News from the Nursing World—** Our Christmas Distribution of Clothing—Princess Louise at Glasgow—The Imperial Military Nursing Service—The Secretary for War and Poor-law Nurses—Discharge of a Nurse at Bury—Hackney Infirmary Scandal—A Joke out of Place—The Nurse and the Hospital Committee—A Question of Discipline at Trim—Trained Nurses and Domestic Servants—Workhouse Masters and the Superintendent Nurse—The Amusements of Hospital Nurses—A Good Start at Sheffield—Chicago Visiting Nurses and the Prevention of Tuberculosis—Operatic Performances and District Nursing—Social Gathering in Somerset—Short Items - - - - - 173-176
**The Nursing Outlook** - - - - - - - - 177
**Lectures on Ophthalmic Nursing** - - - - - - 178
**Our Christmas Distribution** - - - - - - - 179
**Dot and Tiny** - - - - - - - - - - - - - 179
**The Work of a Small Provincial Hospital** - - 180
**Travel Notes and Queries** - - - - - - - - 181
**The Nurses' Bookshelf** - - - - - - - - 181
**Everybody's Opinion** - - - - - - - - - 182
**Novelties for Nurses** - - - - - - - - - 183
**Appointments** - - - - - - - - - - - 183
**Presentations** - - - - - - - - - - - 183
**"The Hospital" Convalescent Fund** - - - - - 183
**Echoes from the Outside World** - - - - - - 184
**A Book and its Story** - - - - - - - - - 185
**Notes and Queries** - - - - - - - - - - 186
**For Reading to the Sick** - - - - - - - - 186

